# The association between *XPG* polymorphisms and cancer susceptibility: Evidence from observational studies

**DOI:** 10.1097/MD.0000000000007467

**Published:** 2017-08-11

**Authors:** Cuihong Han, Xiaoyi Huang, Ruixi Hua, Shujie Song, Lihua Lyu, Na Ta, Jinhong Zhu, Peixi Zhang

**Affiliations:** a Department of Pathology, Jining No. 1 People's Hospital, Jining, Shandong; b Department of Pathology, Changhai Hospital, Second Military Medical University, Shanghai; c Department of Oncology, The First Affiliated Hospital of Sun Yat-sen University, Guangzhou, Guangdong; d Zhejiang Provincial Key Laboratory of Medical Genetics, Wenzhou Medical University, Wenzhou, Zhejiang; e Molecular Epidemiology Laboratory, Department of Laboratory Medicine, Harbin Medical University Cancer Hospital, Harbin, Heilongjiang; f Department of Cardio-Thoracic Surgery, Jining No. 1 People's Hospital, Jining, Shandong, China.

**Keywords:** cancer, meta-analysis, polymorphism, risk, XPG

## Abstract

**Background:**

Exposure to environmental carcinogens can cause damages to DNA. If not properly repaired, the DNA damages may increase the risk of carcinogenesis. *Xeroderma pigmentosum group G* (*XPG)* gene is an essential gene in the nucleotide excision repair (NER) pathway. The association between *XPG* polymorphisms and cancer susceptibility has been the focus of attention in the molecular epidemiology of cancer. However, the conclusions have been divergent. Therefore, we conducted a comprehensive meta-analysis to precisely evaluate the association of 3 frequently investigated *XPG* polymorphisms (rs751402, rs873601, and rs2296147) with cancer risk.

**Methods:**

*Pubmed*, *EMBASE*, and Chinese National Knowledge Infrastructure (*CNKI*) were searched for relevant studies in English and Chinese. Odds ratio (OR) and 95% confidence interval (CI) were used to assess the association between *XPG* polymorphisms (rs751402, rs873601, and rs2296147) and cancer risk.

**Results:**

Twenty-three studies were included. Overall, there was no significant association between rs751402 polymorphism and overall cancer risk under the 5 gene models. However, we observed strong correlation between rs751402 polymorphism and gastric cancer (C vs T: OR=1.21, 95% CI = 1.00–1.26, *P* = .045; TC vs CC: OR = 1.12, 95% CI = 1.00–1.24, *P* = .041; TC/TT vs CC: OR = 1.13, 95% CI = 1.02–1.26, *P* = .020). There was a significant correlation between rs873601 polymorphism and cancer risk under the homozygous model (GG vs AA: OR = 1.16, 95% CI = 1.07–1.26, *P* = .001). Moreover, significant association with breast cancer was detected for rs873601 polymorphism under the allele contrast model (G vs A: OR = 1.10, 95% CI = 1.02–1.20, *P* = .021). In the subgroup of Asian, rs873601 polymorphism was related to the susceptibility to cancer (G vs A: OR = 1.07, 95% CI = 1.03–1.12, *P* = .010; GG vs AA: OR = 1.15, 95% CI = 1.06–1.26, *P* = .001; AG/AA vs GG: OR = 1.08, 95% CI = 1.01–1.15, *P* = .031; AA vs AG/GG: OR = 1.13, 95% CI = 1.05–1.21, *P* = .001). Significant association between rs2296147 polymorphism and cancer risk were observed in Asian population (CT vs TT: OR = 0.93, 95% CI = 0.87–0.99, *P* = .036).

**Conclusions:**

Our meta-analysis suggested that the rs873601 polymorphism was significantly associated with overall cancer risk. The moderate effects of rs751402 and rs2296147 polymorphism on cancer susceptibility might be highly dependent on cancer type and ethnicity, respectively. Large studies are needed to validate our findings, especially in Caucasian and African population.

## 1 Introduction

To date, cancer remains a major health problem around the world with enormous health expenditure.^[1]^ Cancer is a complex disease linked to environmental factors, hereditary susceptibility, and gene–environment interactions. Environmental carcinogens exposure can cause DNA damage, which may increase the risk of developing cancer, if not restored properly. In addition, during the process of DNA replication, DNA mismatch may occur and lead to genome instability.^[2]^ In human, the DNA repaired mechanism is a complex biological process, including several pathways and numerous proteins. If DNA damage cannot be properly and efficiently restored due to defects in DNA repair pathways, the risk of cancer could be elevated by many folds.

DNA damages caused by environment factors, such as ultraviolet (UV) radiation, tobacco, and diet, are closely related to human disease. DNA repair pathways play an important role in maintain genomic integrity and stability. There are 5 major DNA repair pathways, homologous recombinational repair (HRR), nonhomologous end joining (NHEJ), nucleotide excision repair (NER), base excision repair (BER), and mismatch repair (MMR).^[3,4]^ Among these pathways, NER removes helix-distorting DNA lesions and structures from the genome with 4 steps: lesion recognition, protein binding, oligonucleotide excision, and DNA fragment synthesis.^[5]^ Gene mutation in the NER pathway can lead to several diseases in human, including xeroderma pigmentosum (XP).^[6]^ XP is a prototypical DNA disorder featured by sensitivity to UV radiation and extremely increased susceptibility to skin cancer.^[7]^ The XP patients can be classified as 8 complementation groups, XPA through XPG.

The *xeroderma pigmentosum group G* (*XPG*) gene, also termed as excision repair cross-complementation group 5 (*ERCC5*) gene, is an essential gene in NER pathway. It encodes a structure-specific endonuclease that has multiple function during the NER.^[8]^ Previous studies indicated that *XPG* polymorphisms can influence the DNA repair ability for tobacco and alcohol-induced DNA damage, thereby increasing the susceptibility to cancer.^[9,10]^ For instances, Lu et al reported that *XPG* rs17655 polymorphism contributed to increased risk of laryngeal cancer^[11]^; He et al^[12]^ indicated that another polymorphism in *XPG* gene, rs2094258, might be associated with neuroblastoma susceptibility. Moreover, a meta-analysis provided the evidence of the association between *XPG* rs17655 polymorphism and colorectal cancer risk.^[13]^ However, overall, the association between *XPG* polymorphisms and the risk of cancer remains conflicting. Therefore, it is necessary to conduct a comprehensive to reevaluate the association of interest.

## 2 Materials and methods

### 2.1 Literature search strategy

We searched *Pubmed*, *EMBASE*, and Chinese National Knowledge Infrastructure (*CNKI*) for relevant studies written in English and Chinese. The key words were as follows: “*xeroderma pigmentosum group G* or *XPG* or *excision repair cross-complementation group 5* or *ERCC5* or rs751402 or rs873601 or rs3742282 or 2296147 OR rs17326289,” “polymorphisms or SNP or variation,” and “carcinoma or tumor or neoplasm or cancer.” In addition, the references list of included studies and relevant reviews were manually searched for eligible studies. The last search was performed on December 30, 2016.

### 2.2 Inclusion and exclusion criteria

Studies were included based on the following criteria: studies investigating the association between the *XPG* polymorphisms and the risk of cancer; case–control studies; articles providing genotype distribution data or corresponding odds ratio (OR) and 95% confidence interval (CI); and articles published in English or Chinese. The exclusion criteria were as follows: letters, editorials, reviews, meta-analysis, or systematic reviews; published studies containing duplicate data; studies lacking of genotype distribution data or corresponding OR and 95% CI; and not case–control study or cohort study.

### 2.3 Study selection and data extraction

Eligible studies were selected by 2 researchers independently according to the inclusion and exclusion criteria. All disagreements were discussed. All the researchers would vote if a consensus could not be reached. All the information was double checked by authors who were responsible for data extraction. The following data were extracted from the original studies: author, publish year, country or region, ethnicity, cancer types, sample size including case and control sample size, genotype data of rs751402, rs873601, and rs2296147, and *P* value of Hardy–Weinberg Equilibrium (HWE). The meta-analysis was approved by the Hospital Ethics Committee (Jining No. 1 People's Hospital) to not reidentify the participants.

### 2.4 Statistical analysis

The HWE in control subjects in all the articles was assessed by χ^2^ test. OR and 95% CI were used to estimate the association between *XPG* polymorphisms and cancer susceptibility. Pooled analysis was performed under the 5 genotype models, including allele contrast, homozygous, heterozygous, dominant, and recessive models. Chi-square-based *Q* statistic test was used to estimate the between-study heterogeneity via *I*^2^ value and *P* value. If *I*^2^ > 50%, random effects model was applied to calculate the pooled OR and 95% CI. Otherwise, fixed-effects model was adopted. Moreover, subgroup analyses were conducted by cancer type and ethnicity. All the *P* values were 2-sided. We referred to statistically significant as *P* < .05. Sensitivity analysis was applied to assess the stability of the results by sequentially removing one study at a time and recalculating OR and 95% CI. Funnel plot and Egger linear regression test were performed to estimate the publish bias. Asymmetrical funnel plot or *P* < .1 indicated that publication bias was significant. STATA 12.0 (STATA Corporation, College Station, TX) was applied to perform all the statistical analysis.

## 3 Results

### 3.1 Search results and study characteristics

**Figure 1:**
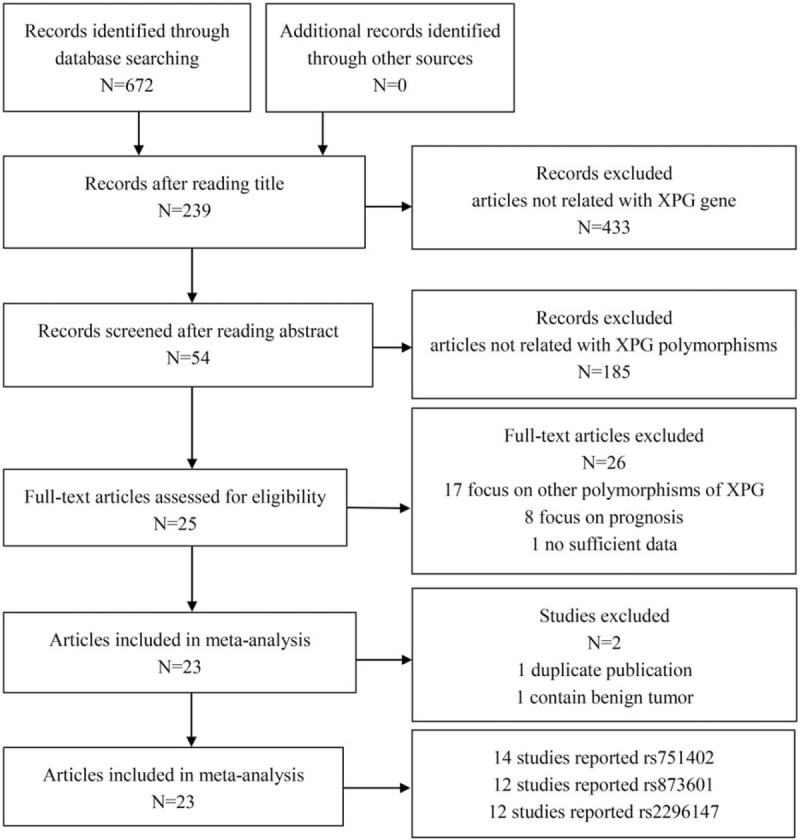
The flow diagram of study selection and inclusion process.

Based on the inclusion and exclusion criteria, 23 eligible articles were identified from 672 potential publications (Fig. 1). Among these eligible studies, there were 14 studies with 7291 cases and 7889 controls focusing on rs751402; 12 articles with 9158 cases and 10,073 controls on rs873601; and 12 articles with 9288 cases and 9863 controls on rs2296147. The basic characteristics of the included studies on *XPG* polymorphisms (rs751402, rs873601, and rs2296147) and cancer risk were summarized in Table 1. Out of the 23 articles, 8 articles focused on gastric cancer, 4 articles on breast cancer, 3 articles on squamous cell carcinoma, 2 articles on hepatocellular carcinoma, and 1 article on each of endometrial cancer, prostate cancer, pancreatic cancer, colorectal cancer, and neuroblastoma. As for ethnicity, 21 studies were conducted among Asian population, 1 article among Caucasian, and 1 article among the mixed population.

**Table 1:**
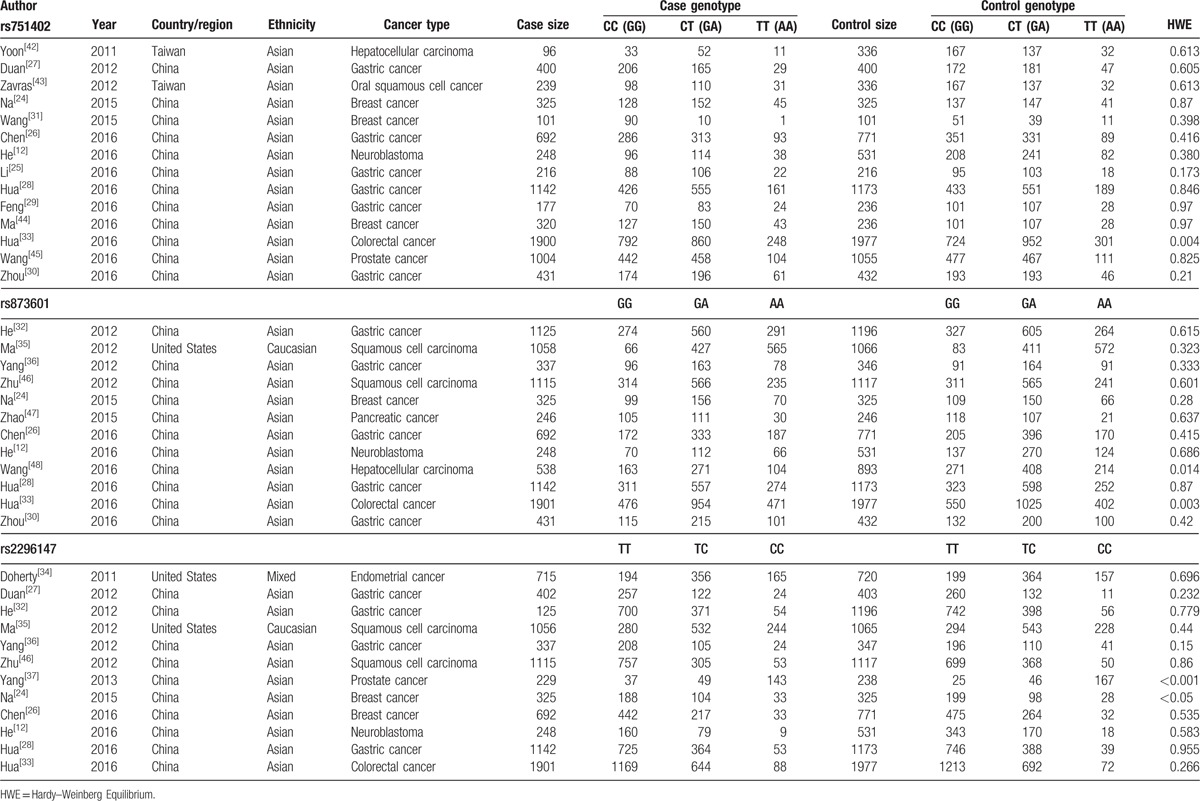
The basic characteristics of the included studies on XPG polymorphisms and cancer risk.

### 3.2 Meta-analysis results

**Figure 2:**
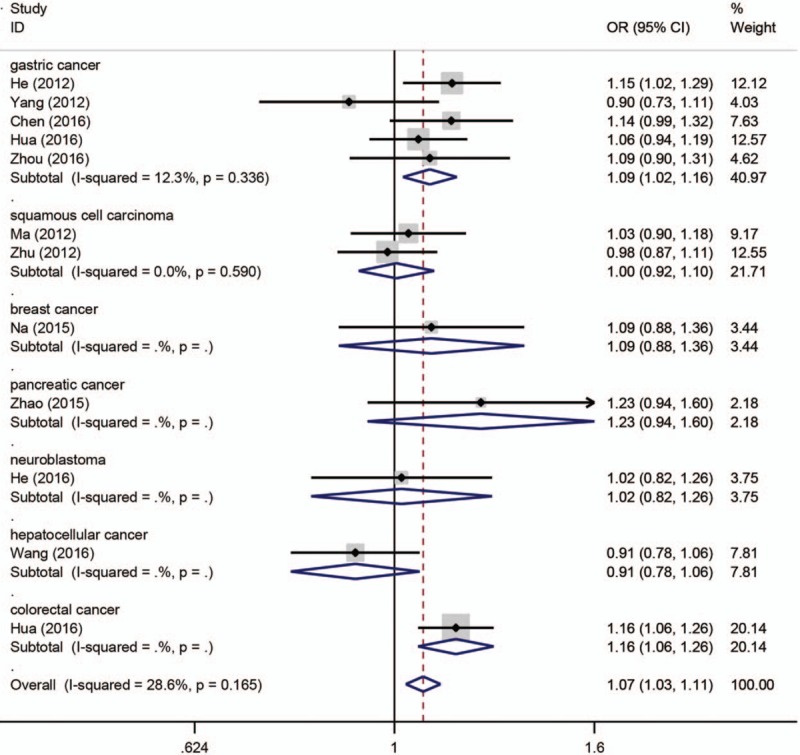
Forest plot of cancer risk related with XPG rs873601 polymorphism stratified by cancer type in allele model.

**Figure 3:**
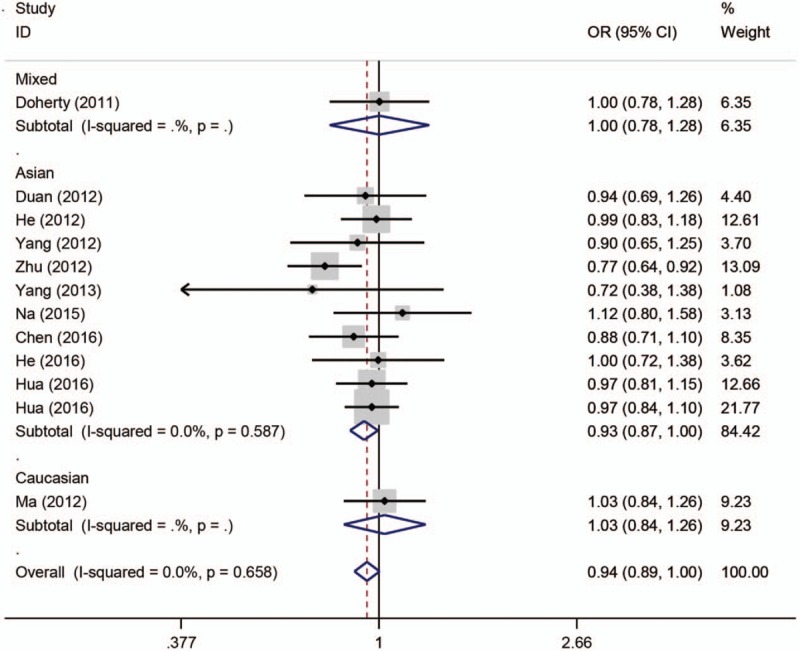
Forest plot of cancer risk related with XPG rs2296147 polymorphism stratified by ethnicity under the heterozygous model.

The main results of pooled ORs and 95% CIs between rs751402, rs873601, and rs2296147 polymorphisms and cancer risk are showed in Table 2, Table 3 and Table 4, respectively. Overall, there is no association with cancer observed for the studied rs751402 polymorphism in 5 gene model. Stratified analysis by cancer type revealed that there were strong correlation between rs751402 polymorphism and gastric cancer (C vs T: OR = 1.21, 95% CI = 1.00–1.26, *P* = .045; TC vs CC: OR = 1.12, 95% CI = 1.00–1.24, *P* = .041; TC/TT vs CC: OR = 1.13, 95% CI = 1.02–1.26, *P* = .020). Stratified analysis by ethnicity indicated there is an association between rs873601 polymorphism and cancer risk in Asian population (G vs A: OR = 1.07, 95% CI = 1.03–1.12, *P* = .010; GG vs AA: OR = 1.15, 95% CI = 1.06–1.26, *P* = .001; AG/AA vs GG: OR = 1.08, 95% CI = 1.01–1.15, *P* = .031; AA vs AG/GG: OR = 1.13, 95% CI = 1.05–1.21, *P* = .001). However, significant association between rs873601 polymorphism and gastric cancer risk were observed under the allele contrast model (G vs A: OR = 1.08, 95% CI = 1.02–1.16, *P* = .010) (Fig. 2). Moreover, there was a significant association between rs2296147 polymorphism and cancer risk in Asian population under the heterozygous model (CT vs TT: OR = 0.93, 95% CI = 0.87–1.00, *P* = .036) (Fig. 3). In addition, the results suggested that no correlation were found between rs2296147 polymorphism and any of cancer types.

**Table 2:**
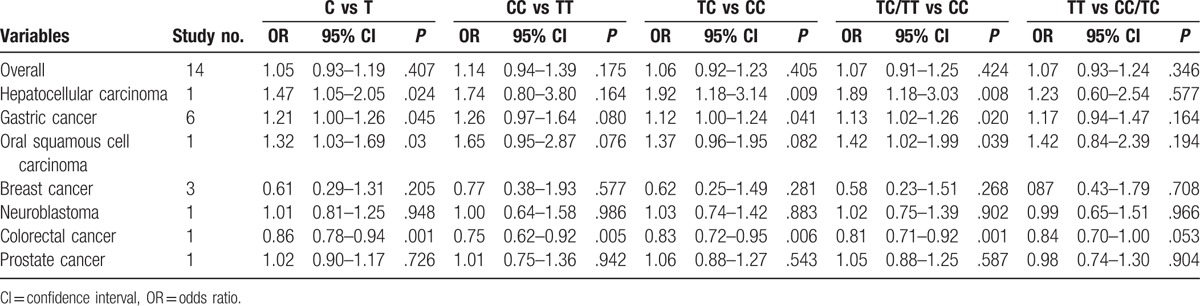
Meta-analysis of rs751402 polymorphism and cancer risk.

**Table 3:**
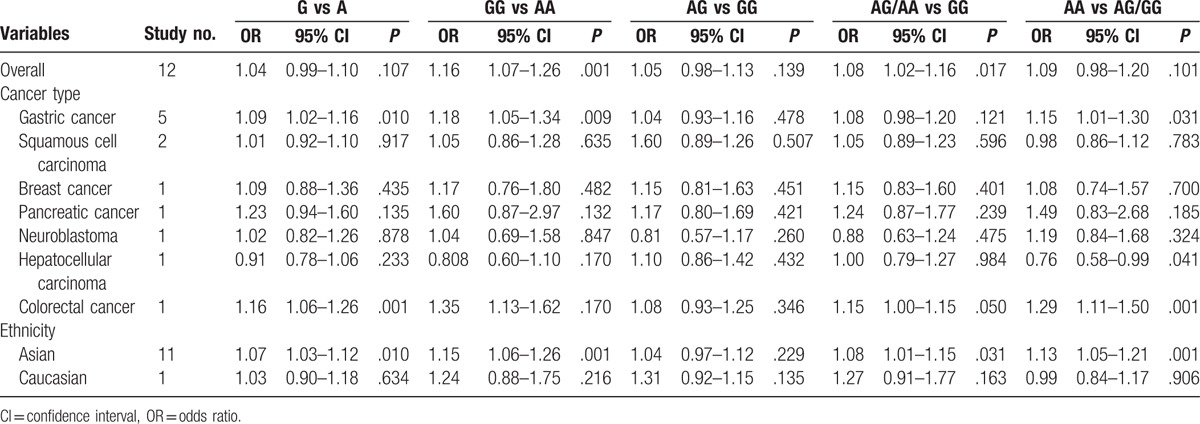
Meta-analysis of rs873601 polymorphism and cancer risk.

**Table 4:**
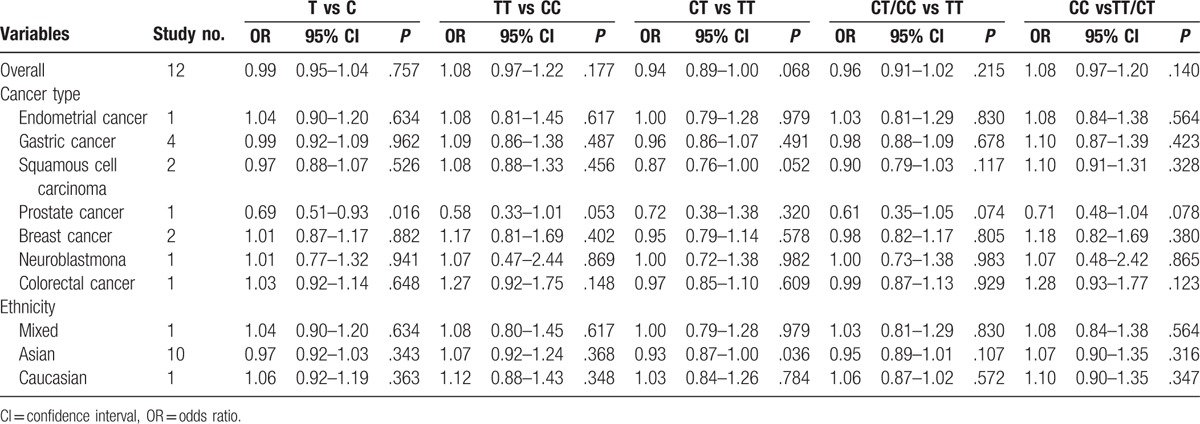
Meta-analysis of rs2296147 polymorphism and cancer risk.

### 3.3 Sensitive analysis and publication bias

We conducted the sensitive analysis and the results suggested that no single study could significantly change the pooled risk estimates, which indicated our meta-analysis results were robust and stable. The Begg funnel plot and Eagger regression test were used to assess the publication bias. The results of Egger test suggested no significant publication bias in our meta-analysis (rs751402: *P* = .537; .162; .617; .550; and .224 for allele contrast, homozygous, heterozygous, dominant, and recessive models, respectively; rs873601: *P* = .380; .450; .690; .841, and .555 for allele contrast, homozygous, heterozygous, dominant, and recessive models, respectively; rs2296147: *P* = .248; .707; .871; .486, and 0.914 for allele contrast, homozygous, heterozygous, dominant, and recessive models, respectively).

## 4 Discussion

XPG protein serves as structure-specific endonuclease that cleaves several kinds of substrates with ss/ds DNA junction in NER.^[14]^ During the process of NER, XPG participates in both global genome repair (GG-NER) and transcription-coupled repair (TC-NER). In GG-NER, XPG interacts with transcription factor II H (TFIIH) and this interaction leads to the recruitment of XPG to NER complex at the sites of DNA damage caused by UV radiation.^[15,16]^ Then the NER complex including XPG removes the lesion from DNA strand. Several investigations indicated that XPG is related to carcinogenesis and prognosis of various types of cancer.^[17–19]^ Notably, genetic variations in the *XPG* gene show significant association with the risk of various cancers.^[20–22]^ In addition, some meta-analysis focusing on one of *XPG* polymorphisms and cancer risk revealed that *XPG* rs17655 contributed to cancer susceptibility.^[13,23]^ However, the association between other polymorphisms and cancer risk remains controversial.

To the best of our knowledge, this is the first meta-analysis that focused on *XPG* polymorphisms (rs751402, rs873601, and rs2296147) and cancer risk. Pooled analysis indicated that there was no significant association of *XPG* gene polymorphisms rs751402 and rs2296147 with cancer risk individually. However, there is association with cancer observed for the studied rs873601 polymorphism under the homozygous model. In the stratified analysis of Asian subgroup, there is a significant association between rs873601 polymorphism and cancer risk under the allele contrast, homozygous, dominant, and recessive models. The rs2296147 polymorphism was significantly associated with the decreased cancer risk in Asian population under the heterozygous model.

The results of current meta-analysis suggest that the rs751402 polymorphism was not associated with overall cancer risk, which is coincided with previous studies.^[12,24–26]^ In subgroup analysis by cancer type, there were strong correlation between rs751402 polymorphism and gastric cancer, while no relationship between rs751404 polymorphism and breast cancer was observed. The association with gastric cancer risk was based on 6 case–control studies, and our results were in accordance with Duan et al.^[27]^ However, the results were inconsistent with other studies, which failed to find significant association between rs751402 polymorphisms and gastric cancer.^[28–30]^ Zhou et al found that TT genotype was associated with an increasing risk of gastric cardia adenocarcinoma.^[30]^ The controversial results might be partially due to the variations in the sample sizes and the control group sampling methods. In Duan et al’ studies, the sample size was relatively small, and the healthy controls were patients with mild superficial gastritis receiving endoscopic examination. And the selection bias in controls might be the major reason leading to the discrepancy.

The meta-analysis indicated that no significant association was found between the rs751402 and breast cancer risk. Na et al conducted a hospital-based case–control study to explore the association between *ERCC5* gene polymorphism and found that rs751402 might not be the risk factor for breast cancer.^[24]^ Wang et al conducted another hospital-based case–control study in 2015 with 101 cases and 101 controls. In contrast, they reported that rs751402 was a strong protective factor against breast cancer.^[31]^

With regard to rs873601 polymorphism, there was a significant association with total cancer risk observed under the homozygous model. When stratified by cancer type, rs873601 polymorphism was shown to significantly increase gastric cancer under the allele contrast model. This result was in accordance with the findings by He et al.^[32]^ In addition, Chen et al found that *XPG* rs873601 polymorphism was associated with gastric cancer susceptibility under the recessive model.^[26]^ As for ethnicity, 11 of 12 articles were performed in Asian population and only one article was conducted in Caucasian population. A significant association with cancer susceptibility was found in Asian population under the allele contrast, homozygous, dominant and recessive models. The results were consistent with those of previous studies.^[26,33]^ However, other studies could not find any correlation between rs873601 polymorphism and cancer risk. This discrepancy may result from many factors, including the study designs, genotyping methods and sources of control and cases. Currently, the association between rs873601 polymorphism and cancer risk is mainly investigated among Asian population, while few studies conducted in other population such as Caucasian and African.

Similar to previous studies,^[12,24,26,32,34,35]^ our meta-analysis results indicated that the rs2296147 polymorphism might not contribute to cancer susceptibility. However, Yang et al investigated the association between *XPG* gene polymorphisms and gastric cancer risk and found that rs2296147 CC genotype was related to increased risk of gastric cancer.^[36]^ In this study, the control group was recruited from other surgical department and selection bias might exist. Another study focusing on rs2296147 polymorphism and prostate cancer risk was conducted by Yang et al. They suggested that rs2296147 polymorphism T allele was strongly associated with prostate cancer risk.^[37]^ In this study, the sample size was relatively small with only 241 prostate cancer cases and 264 healthy controls. Na et al found that rs2296147 showed no association with breast cancer risk, but the genotype distribution was not in agreement with HWE. As for ethnicity, there was significant relationship between rs2296147 polymorphism and cancer risk in Asian population in heterozygous model. This result suggested that rs2296147 may be a protective factor against carcinogenesis in Asian population.

As we know, cancer can occur in the different sites of the body. Although cancer is a highly heterogeneous disease, different types of cancer share some similar mechanisms and pathogenesis. In 2011, Hanahan and Weinberg^[38]^ have summarize several hallmarks of cancer, including sustaining proliferative signaling, evading growth suppressors, avoiding immune destruction, enabling replicative immortality, tumor promoting inflammation, activating invasion and metastasis, inducing angiogenesis, genome instability and mutation, resisting cell death, and deregulating cellular energetics. Apart from common hallmarks, every type of cancer has its unique biological characteristics. Previous studies reported that *XPG* gene polymorphisms may affect *XPG* gene expression, leading to genomic instability and carcinogenesis.

As *XPG* polymorphisms are new genetic variations discovered recent years, the relationship between these SNPs and cancer risk is not clear. Thus, it is necessary to clarify such association. There are several meta-analysis focused on other *XPG* polymorphisms and cancer risk previously. Ding et al conducted a meta-analysis of the association between *XPG* Asp1104His polymorphism and breast cancer risk.^[39]^ This meta-analysis included 10 studies with 5235 cases and 5685 controls, but failed to find any significant association. Zhu et al reported that *XPG* Asp1104His polymorphism might have no correlation with susceptibility to overall cancer risk in a meta-analysis with 23,490 cases and 27,168 controls.^[23]^ Another meta-analysis investigating the association between *XPG* Asp1104His polymorphism and cancer risk suggested that *XPG* Asp1104His polymorphism may be a low-penetrant risk factor in some kinds of cancer.^[40]^ Jiang et al suggested that *XPG* Asp1104His polymorphism may increase the risk of head and neck cancer in a meta-analysis of 8 case–control studies.^[41]^ All these meta-analysis focused on *XPG* Asp1104His polymorphism and cancer risk.

Some limitations might exist in this study should be mentioned. First, the between-study heterogeneity was relatively large. This may be due to variations in the sample sizes, the genotyping methods or the selections of case among the original studies. Second, not all the studies included in our meta-analysis were in accordance with HWE. When we omitted this study deviated from HWE to recalculate risk estimates, the results were not altered substantially, suggesting that the results of our meta-analysis were reliable. Third, the control groups of some original studies were enrolled from other patients without malignant tumors, which may be a source of selection bias.

In conclusion, the results of our meta-analysis suggested that there was no any significant association between rs751402 and rs2296147 polymorphisms and overall cancer risk. There was a significant correlation between rs873601 polymorphism and cancer risk in homozygous model. The rs873601 polymorphism was shown to increase gastric cancer risk. In the subgroup of Asian, rs873601 polymorphism was related to the susceptibility of cancer. Moreover, there was significant relationship between rs2296147 polymorphism and cancer risk in Asian population. Additional large studies should be conducted to explore the association between *XPG* gene polymorphisms and cancer risk in the future.
